# A Risk-Based Permitting Process for the Managed Movement of Animals and Products of Animal Origin as a Tool for Disease Management

**DOI:** 10.3389/fvets.2019.00433

**Published:** 2019-12-03

**Authors:** Jamie Umber, Marie Culhane, Carol Cardona, Timothy Goldsmith

**Affiliations:** ^1^Secure Food Systems Team, University of Minnesota, St. Paul, MN, United States; ^2^Department of Veterinary and Biomedical Sciences, College of Veterinary Medicine, University of Minnesota, St. Paul, MN, United States; ^3^Department of Veterinary Population Medicine, College of Veterinary Medicine, University of Minnesota, St. Paul, MN, United States

**Keywords:** permitting, permitted movement, risk assessment, managed movement, foreign animal disease, disease outbreaks, continuity of business

## Abstract

During a foreign animal disease (FAD) outbreak, in addition to detecting, controlling, containing, and eradicating the FAD, one of the goals of response in the United States (US), and many other countries, is to allow the managed movement of non-infected animals and non-contaminated animal products from within FAD control areas to facilitate continuity of business (COB). Permits issued by government authorities are the mechanism by which such managed movements are allowed in the US, resulting in permitted movements. The overall purpose of issuing permits during an outbreak is to minimize the risk of disease spread while still allowing movement of products or animals; thus, the risk associated with each permitted movement must be considered. Currently, there are federal guidelines for the various permit types and purposes. These guidelines state that permits should be “based on science and risk-based information.” However, federal guidelines with specific procedures to determine risk are not readily available nor do they explicitly enumerate measures to assist regulatory authorities in using risk to guide decisions to grant permitted movement or deny a request to move. Although some pro-active risk assessments (RAs) have been conducted to determine risk of moving certain animals and their products, there will always be animal and product movements for which no pro-active RAs exist. We present here a process description of steps to conduct risk-based permitting with appropriate resource allocation to permitting by industry and regulatory authorities during an FAD outbreak.

## Introduction

During a foreign animal disease (FAD) outbreak in the United States (US), the goals of response include not only detecting, controlling, and eradicating the FAD but also continuity of business (COB) for companies and farms with non-infected animals and non-contaminated animal products (1). Key among the many critical activities required to achieve these goals simultaneously, are quarantine and movement controls for animals and premises at highest risk of disease infection and/or spread. Written permits issued by responsible regulatory authorities are a mechanism by which movement controls can be implemented, resulting in permitted movements. The process by which permits are managed (i.e., submitted, reviewed, issued or denied, recorded, and tracked) is called “permitting” in the US. Other countries appear to have comparable approaches—requiring written, or otherwise designated, competent authority approval—to managed or controlled movements during animal disease outbreaks, though specific “permitting” terminology is lacking [for example, see European Commission Council Directives and Commission Decisions for control of various diseases and the Zoning and Compartmentalization Chapter of the World Organization for Animal Health (OIE) Terrestrial Animal Health Code] (2, 3). A key objective of permitting is to allow for movements that are unlikely to spread disease, based on scientific evidence, and that prevent unintended consequences of movement controls (e.g., overcrowding; depopulation and disposal of animals that are not infected)—in other words, permitting should decrease risk. In this context, permitting approaches risk as a combination of the epidemiological probability of an event (i.e., movement results in disease spread) and the consequences of the event (i.e., consequences of disease spread).

The US Department of Agriculture (USDA) FAD Preparedness and Response (FAD PReP) Manual for Permitted Movement explicitly states that “permits and associated requirements should be based on science and risk-based information” (4). However, federal guidelines with specific procedures to determine risk are not readily available nor do they explicitly enumerate measures to assist regulatory authorities in using risk to guide decisions to grant or deny a permitted movement. Similarly, specific published procedural guidelines could not be found for other countries known to utilize movement controls during disease outbreaks. Although some pro-active risk assessments (RAs) have been conducted that evaluate the risk of moving certain animals and their products in specific outbreak scenarios, there will always be animals and product movements that need to occur but no pro-active RAs exist. Additionally, the guidance on how to apply the process of permitting and what the roles and responsibilities are for industry and regulatory officials is not intuitive. We present here two processes to facilitate COB: (1) using a risk-based approach to guide permitting decisions for animal and animal product movements, and (2) appropriate resource allocation by industry and regulatory authorities to permitting during an FAD outbreak. These concepts can be translated further by regulatory authorities into individualized state permitting plans or perhaps for incorporation into national emergency response plans in countries beyond the US.

## Existing Guidelines for Permitted Movement in the US

The USDA Animal and Plant Health Inspection Service Veterinary Services (APHIS VS) has broad authority over interstate movements and the ability to intervene during FAD incidents in the US^1^. However, states have primary authority over intrastate movements as well as requirements for entry into their respective state. As a result, each state may have a unique system and criteria for allowing permitted movement in an outbreak situation. Regional approaches that help coordinate permitting across borders may therefore have great value for areas with interconnected agricultural systems.

The FAD PReP Manual for Permitted Movement provides broad guidance on permit types and premises descriptions related to managed movement, as well as a process for using the Emergency Management Response System 2.0 (EMRS2) for the requesting, review, and approval of permits and also data management for traceability (4). The specific permit guidance and mitigation criteria that address the risk of specific animal and product movements are not available for all situations. Nor does a pre-defined process exist that delineates how roles and responsibilities for permitting are to be carried out by industry and regulatory officials.

In general, it is the responsibility of the producer to ensure that the criteria for permitted movements (e.g., specific biosecurity, diagnostics, etc.) are met and documented before the movement occurs. The originating and destination states have the discretion to then validate or check that these criteria satisfy the state's particular needs (4). Specific to COB movements, several detailed permit guidances based on pro-active RAs are available as part of existing Secure Food Supply (SFS) plans (6).

## Experience From Previous Outbreaks

Movement controls have had a role in effective outbreak control in multiple outbreaks (7–10). Valuable experience has been gained from previous large outbreaks in particular that have necessitated high-throughput operations for activities such as permitting, managed movements, and laboratory testing (8, 11). During the 2014/2015 highly pathogenic avian influenza (HPAI) outbreak, over 7,500 permits were issued for approximately 20,000 movements and these managed movements were accomplished without spreading disease (8, 12). However, this high-throughput significantly strained industry and government resources and likely would not have been sustainable had the outbreak continued (8, 13). Comparatively, avian influenza outbreaks in 2016 and 2017 were more limited in size and scope and did not necessitate such high-throughput permitting operations (9, 10).

High-throughput needs during any large FAD outbreak can consume staff resources unless a process for delegating responsibilities to both industry and regulatory representatives is used. Previous outbreaks have shown how the want and need to stop the spread of disease are equal for impacted commercial agriculture industries and regulatory officials. Yet, despite this common desire, it is the agricultural industry that has more immediate knowledge of activities occurring on farms that may expand or reduce an outbreak. Importantly, industry partners also have the ability to implement risk mitigation measures and do so for other pathogens daily. Our proposed risk-based permitting process builds on these experiences and is founded in the public-private partnerships that grew from the 2014–2015 HPAI outbreak.

## Proposed Risk-Based Permitting Process

The risk-based permitting process can serve as a method for agricultural industries needing business continuity to use during an FAD outbreak, helping build on their knowledge and increase their ability to work with state and federal authorities to inform and perform permitted movements. A functional risk-based permitting process should ensure that, for all permits, risk is considered before a permit to move is approved or denied. This requires an objective understanding of the risk of the movement, including any mitigations that will be used to decrease risk, knowledge that mitigations can and will be applied properly, and an understanding of the context of the move. When these things are known, then the process of risk-based permitting can occur.

A step-wise risk-based permitting process is described below, including roles of responsible parties during each step (see Table 1). While the exact responsible party, down to the specific person, will need to be determined by individual states or responsible regulatory officials, the delegation and communication of roles is a key factor in preparedness. This becomes most clear when considering the management of risks associated with a movement. In order for successful risk mitigation, sufficient resources need to be allocated to the mitigation process. From a permitting perspective, this means that appropriate and sufficient numbers of people need to be available to conduct each step. This necessitates a realistic estimate of availability and capability of both regulatory officials and industry personnel.

**1. Define desired movement:** Defining the movement for which a permit is desired means that all of the information required for a permit request are identified. Specifically, what item will be moved; why the item will be moved; where are the origin and destination premises of the item movement; and when will the move occur (4). Typically, a movement will be defined via a question or actual movement request from industry or from within a unified incident command.^2^ Once the movement is defined, the risk of that movement needs to be evaluated to determine if a permit should be granted and if any specific mitigations and other criteria are needed to address any risk to an acceptable level.
**2. Situational assessment:** Before the resource-demanding process of risk-based permitting moves further, responsible regulatory officials should determine if the circumstances of the current outbreak situation and premises can allow for a potential permitted movement. In some cases, all movements will simply be stopped and the risk-based permitting process ends here. If movements may be possible, depending on the risk posed by the movement, then the risk-based permitting process proceeds to the next steps and the unified incident command refers the movement request or question to a Permitting Advisory Group (see Step 3 text).
**3. Determine if an applicable risk assessment exists:** The process to determine risk takes multiple steps and multiple people. These steps and who will accomplish them are often not included in emergency preparedness plans. Having specific roles for people and positions delineated in state or national plans can speed a permitting process during an outbreak. For example, if an RA for a particular movement already exists then much work can be avoided by simply referencing the RA. However, the permitting authority not only has to ascertain if such an RA exists but also, determine if the existing RA is applicable to the requested movement.

**Table 1:** Proposed steps for risk-based permitting [responsible party/ies included in brackets].

**1. Define desired movement:** what item is to be moved; why is the item to be moved (e.g., moving direct to farm, to landfill, or into commerce); where are the origin and destination premises; and when will the move take place (over what dates). [Industry or unified Incident Command]
**2. Conduct situational assessment:** Responsible regulatory officials determine if the current outbreak situation and premises circumstances can allow for a potential permitted movement. a) If not, process stops here. b) If movement may be possible, proceed to step 3. [Unified Incident Command]
**3. Determine if applicable risk assessment exists:** review existing risk assessments and available guidance. a) If a risk assessment does not exist or is not applicable, move to next step and conduct an *ad hoc* risk assessment. [Permitting Advisory Group]
**4. Determine risk and feasible risk mitigations:** either from an existing applicable risk assessment or during an *ad hoc* risk assessment process, identify feasible risk mitigations (i.e., permit guidance/criteria) for the movement and determine the final risk rating for the movement. [Permitting Advisory Group]
**5. Determine acceptability of movement given final risk:** responsible regulatory officials consider situation/outbreak circumstances to determine if a movement with the given risk level (identified during the previous step 4) is acceptable. [Permitting Authority]
**6. Allocate resources:** delegate responsibilities for oversight and communication of movement requirements to appropriate personnel based on risk. [Industry and Unified Incident Command]

Creation of a Permitting Advisory Group (PAG) will assist in both determining if an applicable RA exists and also determining a final risk rating (the next step in the process, Step 4) for a requested movement. The PAG ideally comprises individuals with expertise regarding the disease, the specific commodity, the industry, RA, outbreak circumstances, and regulatory requirements. The PAG may include additional participants depending on the specific movement or outbreak in question. The subject matter experts of the PAG will be able to assist in locating and reviewing any existing RA and any associated guidance. Close communication and collaboration among individuals of the PAG—with an outlet for rapid, up-to-date communication with the unified Incident Command—is needed to evaluate existing RAs for their applicability to the outbreak.

While having an RA ready for use at the outset of an outbreak can greatly assist a risk-based permitting process, pro-active RAs are based on many assumptions. If not all assumptions are met for a particular premises or situation, then the overall risk conclusion of a pre-existing RA may not be applicable to the desired move, even if the disease and commodity are the same. A specific example where applicability may be a concern is with assumed mitigation measures. For instance, the Pre-Movement Isolation Period (PMIP) is a mitigation measure intended to reduce the risk of disease exposure on a premises in the days leading up to animal movement in order to increase the likelihood of disease detection pre-movement. For some existing poultry RAs (e.g., birds to market or pullets off a farm), it is assumed that a PMIP is in place for a certain number of days pre-movement (15, 16). However, in some situations, like at the outset of an outbreak or immediately after a new Control Area is established, a full PMIP may not have been implemented for a premises requesting a permit. In that case, the risk rating will not be accurate and in fact, the risk may be much higher. In such a situation, the permitting authority would need to weigh the immediacy of the need for the move, the feasibility of waiting the full PMIP, and the potential to expand the outbreak by approving the move as is. Again, review of existing RAs and their applicability necessitates knowledge of the outbreak situation and industry circumstances specific to the premises in question.

**4. Determine risk and mitigation measures:** In addition to providing insight into the likely risk of particular movements, pro-active RAs also have the benefit of elucidating feasible measures that can mitigate risk. In the process of reviewing an existing RA, these mitigation measures can be compiled into permitting guidances or permitting criteria that must be implemented to achieve the risk level indicated in the RA (6). Risk assessments may even include supplemental information that could be considered on a case-by-case basis to lower risk levels (16).

If an existing RA is not applicable or if none exist, then, to move forward with risk-based permitting, risk will need to be evaluated for the particular movement and circumstances. This could be accomplished via an *ad hoc* risk assessment or similar science- and risk-based evaluation (4). The need for *ad hoc* RA will arise in every outbreak since there is always a level of uncertainty about the nature of the next outbreak, what pathogens will be involved, and what commodities will be affected. Further, agricultural industries are constantly changing and the processes used to create and move products are in constant flux. Because changes impact how activities, like biosecurity, happen, they also impact the risk of those activities. Full RAs take significant time (months to years) to complete and usually include both quantitative and qualitative analyses. Quite often, the proactive full RA estimations report likelihood ratings based on a six-level scale, specifically negligible, very low, low, moderate, high, and extremely high. An abbreviated *ad hoc* process, on the other hand, can be completed in a much shorter timeframe than a full RA. It is important to note, however, that the *ad hoc* process is based on the best available information, not necessarily all information. Furthermore, since the process is shortened, there will be a higher degree of unknown risk. Thus, the levels of uncertainty surrounding risk as a result of the *ad hoc* process must be included in the final consideration. While the detailed methods for an *ad hoc* RA are beyond the scope of this paper, we propose that the same PAG identify and consider risk pathways, detection methods, and mitigation strategies to evaluate overall movement risk and to reduce the unknown factor by defining permit specific conditions and criteria needed.

**5. Determine acceptability of movement once the final risk rating is given:** Once a risk rating and mitigation measures are determined, we recommend assigning the defined movement to these categories: (1) negligible/low risk; (2) moderate/high risk; or (3) unknown risk. The negligible/low-risk category can be assigned to those movements that received a likelihood estimation of negligible, low, or very low. Similarly, movements that received a likelihood estimation of moderate or high would be placed in the moderate/high-risk category. This organization by category is intended not to undermine the goals of current policies and procedures for managing all risk that is non-negligible. Rather, the proposed categories here are intended to facilitate resource allocation between industry and regulatory officials with regard to the remaining aspects of the permitting process, in particular, the allocation of resources for direct oversight.

At this stage, once a risk rating for the movement is provided by the PAG, the permitting authority will need to determine whether or not it is acceptable to allow the movement to occur given the risk category. For a movement with a risk that is very high and/or deemed unacceptable for current circumstances, the movement should be denied. For a movement with a risk deemed acceptable for current circumstances, then adequate resources are allocated for oversight of the permitted movement, and the resources allocated should be commensurate with the risk of that movement.

**6. Allocate resources for oversight and communication:** Risk-based permitting requires understanding and allocation of responsibilities by both industry stakeholders and regulatory officials. Transparent communication about the risk-based decisions made, responsibilities, and resource limitations can help increase confidence in and compliance with the process by all stakeholders, leading to success. During highly contagious disease outbreaks, regulatory personnel will be stretched from a resource availability perspective. With the multitude of disease response activities involved with such outbreaks, there is a need to reduce straining resources. Utilizing a risk-based permitting structure that allocates resources based on the likelihood and consequences of disease spread can be a more efficient use of resources, focusing on movements that pose the most risk (Table 2). Specifically, the resources needed for auditing permitting criteria can be allocated according to the risk. Descriptions and communication of resource needs will assist all stakeholders' understanding of how many and what resources will be needed.

**Table 2:** An example of risk-based permitting resource allocation.

**Negligible to low risk movements** • Utilize multiday permits (blanket permits) for movements from one premises to one destination that occur over a period of days. • Permit requestor (industry) manages the criteria and needed surveillance/diagnostic reporting under the permit for the approved time period ° Maintain a Monitored Premises status ° Report any changes in situation • Movement reporting is done at regular intervals to meet traceability needs • Incident Command communicates any change in outbreak situation that may affect premises status • Regulatory officials audit permit criteria requirements at level commensurate with risk ° Negligible risk movements-−1 out of 20 permits ° Low risk movements-−1 of 10 permits **Moderate to high risk movements** • Utilize single movement permits for a single movement from one origin premises to one destination premises • Regulatory officials audit permit criteria requirements at level commensurate with risk ° Moderate risk movements—one out of five permits ° High risk movements—one out of one permits

## Conclusions

When risk is incorporated into the permitting process, there remains the very real possibility that sometimes the risk, regardless of the level, will be considered too great to allow the requested movement to occur. For example, although an existing RA indicates a low likelihood that a large number of HPAI infected pullets would be moved when all Secure Poultry Plan mitigation practices are strictly implemented on a premises (16), if the destination premises for the movement is a large layer complex, then the risk may still be considered unacceptably high due to the high consequence to the layer industry if that pullet movement were allowed and the layers became infected. Conversely, if live animals are to be moved to a single-age premises with no other animals on-site (e.g., pullets to single-age layer premises, growing pigs to an empty finisher, calves to an empty pasture or feedlot) the risk may be acceptable to industry even if there is a chance of moving infected but undetected animals. Thus, even for a requested movement with a non-negligible risk, the permit request may be approved following the risk-based process and communication described above. Additionally, whether a certain level of risk is acceptable also may change as an outbreak progresses. For example, during initial phases of an outbreak, any amount of risk may be considered too high as movements are stopped and quarantines put in place in an effort to rapidly stamp out the disease. However, in later phases of an outbreak, there may be more severe animal welfare impacts to weigh against disease spread risk posed by various movements.

Utilizing a transparent approach that includes regulators and industry in the process of risk-based permitting has definite utility in high-consequence animal disease outbreaks. In order for responsible regulatory officials and industry to accept movements during an outbreak, all stakeholders need to be confident in the entire process. This includes the process of risk evaluation, the mitigation procedures and processes that are followed to address known risks, and the process of managing the movements from within a control area during an outbreak, including communication. Previous large outbreaks have demonstrated how response resources rapidly can be consumed and how resource-intensive the permitting process can be. Utilizing the process prosed here could help decrease demands on limited regulatory resources commensurate with risk. The process proposed here allows regulatory officials to focus more of their efforts on moderate to high-risk movements but it does not remove them entirely from the process for low or negligible risk movements. The goal is to efficiently and effectively balance resource allocation between industry and regulators. Importantly, this process includes specific steps for both industry and regulatory officials to have input into evaluating risk of defined movements and determining whether outbreak-specific circumstances dictate such risk as acceptable or unacceptable to allow permitted movement.

## Author Contributions

All authors listed have made a substantial, direct and intellectual contribution to the work, and approved it for publication.

### Conflict of Interest

The authors declare that the research was conducted in the absence of any commercial or financial relationships that could be construed as a potential conflict of interest.

## Footnotes

^1^If an USDA Extraordinary Emergency Declaration or similar national emergency declaration is made, the USDA (or federal government) then has authority over both intrastate and interstate movement. If no such declaration is made, then the USDA provides the oversight [in coordination with State Animal Health Officials (SAHOs) and the unified Incident Command] for interstate permitted movements and any movement that relates to international trade. For more information on State and Federal authorities, see the NAHEMS Guidelines: Quarantine and Movement Control (5).

^2^Incident Command refers to the organizational element responsible for overall management of the outbreak incident. In the US, this terminology comes from using the Incident Command System (a standardized approach to the command, control, and coordination of on-scene incident management, providing a common hierarchy within which personnel from multiple organizations can be effective) (14). A unified Incident Command is used when more than one agency has incident jurisdiction or when incidents cross political jurisdictions as is common with animal disease outbreaks.

## Abbreviations

COB: continuity of business
EMRS2: emergency management response system 2.0
FAD: foreign animal disease
FAD PReP: FAD preparedness and response
HPAI: highly pathogenic avian influenza
PAG: permitting advisory group
PMIP: pre-movement isolation period
RA: risk assessment(s)
SFS: secure food supply
USDA APHIS VS, United States Department of Agriculture, Animal and Plant Health Inspection Service: Veterinary Services.
